# Cultural Responsiveness and Palliative Care during the COVID-19 Pandemic

**DOI:** 10.1089/pmr.2020.0049

**Published:** 2020-08-20

**Authors:** Arika Patneaude, Jennifer Kett

**Affiliations:** Division of Bioethics and Palliative Care, Treuman Katz Center for Pediatric Bioethics, Seattle Children's Hospital and Research Institute, Seattle, Washington, USA.

**Keywords:** COVID-19, cultural awareness, cultural competence, cultural humility, cultural responsiveness, palliative care

## Abstract

During the COVID-19 pandemic, much has changed in the delivery of palliative care (PC). However, cultural responsiveness remains critical to our mission. It is essential to our aims of identifying individual goals of care and providing relief of suffering for our patients. Cultural responsiveness may also enhance trust and could be a mitigating factor in the staggering health care disparities unmasked during this pandemic. In this study, the authors outline the rationale for renewed focus on this issue, and offer some initial suggestions for how culturally responsive PC can be provided even in this extraordinary time.

## Introduction

This is an unprecedented time for the world, for health care professionals, and for palliative care (PC) providers in particular. It feels as though our typical ways of delivering care have all been altered or suspended in some way—the usually essential components of time, relationship, and face-to-face contact have all dramatically changed. We may be overwhelmed with the number of patients and have very little time for each. We may be encased in personal protective equipment (PPE) at the bedside or “present” only virtually by telephone or computer. We may be communicating with our patients' loved ones who are themselves at a distance—even at the end of life. If we are not doing this difficult work ourselves, we are preparing to do so or are supporting our colleagues who already are. Our conversations as a field may be focused on maintaining the most basic elements of PC in the face of an overwhelming patient surge and staffing crises. However, as we balance the many competing needs as individual providers and together as a community, it is important that we continue to maintain our commitment to cultural responsiveness. Cultural responsiveness^*^ is critical to the mission of PC, even in this extraordinary time.

Cultural responsiveness is a vital component of specialty PC. It is a foundational element in the pursuit of our tandem missions of identifying goals of care and providing relief of suffering for patients. Our determination to understand our patients as individuals, and the cultural contexts in which they live, makes us uniquely effective as we address both of these duties. For example, understanding our patient's deeply held belief in vitalism is likely to alter the way that we approach a particular goals-of-care discussion. Appreciating our patient as a member of a historically marginalized community can help us to be more sensitive and adept in raising difficult issues such as withdrawal of life support. Knowing our patients' perspectives on death and dying may help us to address their spiritual or existential suffering. This approach has always been an important part of PC. Now, our patients may be dying alone, far from family, friends, clergy, prayer, familiar food, rituals, music, and other elements that are essential comforts to so many of us when we are sick and dying. Being isolated from one's family, community, and culture is a source of suffering on its own. PC providers may be able to provide some relief by finding creative ways to reconnect patients to that which is most important to them.

In addition to being helpful in the determination of goals of care and the relief of suffering, attention to culture may also enhance trust between patients, families, and the medical team. Although overwhelmed teams may feel that they simply do not have the time to devote to cultural responsiveness, there is also reason to think that culturally responsive care could, on the whole, actually increase team efficiency. Although we are unable to identify any research that demonstrates a direct link between culturally responsive care and increased patient trust, a number of studies have demonstrated an inverse relationship between patients' perceptions of discrimination and trust in health care providers.^2,3^ It is reasonable to believe that care that is individualized, collaborative, and culturally responsive would augment trust, and care that fails to do these things would diminish trust. Enhancing trust is not only the right thing to do for our patients—it is also efficient. Entrenched conflicts rooted in mistrust are resource depleting—often requiring substantial provider time and energy as well as attention from legal, risk management, ethics, and others. In a time when medical teams are stretched to their limits, preventing conflict by enhancing trust is key. In addition, health care providers are at risk for moral injury as they face difficult triage decisions about which patients will be saved in this crisis. Facing angry mistrustful families is likely to heighten this risk. Time spent attending to culture, thereby enhancing trust and relationship, is time well spent in our opinion, and a failure to do so is a false economy.

Finally, culturally responsive care is imperative as we begin to address the health care disparities that are unmasked and exacerbated by this pandemic. “A large body of published research reveals that racial and ethnic minorities experience a lower quality of health services, and are less likely to receive even routine medical procedures than are white Americans.”^4^ We now know that “Persons who are African American or black are contracting SARS-CoV-2 at higher rates and are more likely to die.”^5^ For example, “In Chicago, more than 50% of COVID-19 cases and nearly 70% of COVID-19 deaths involve black individuals, although blacks make up only 30% of the population.”^5^ Other states have demonstrated similar disparities.^5^ In New York City, “this disproportionate burden is validated again in underrepresented minorities, especially blacks and now Hispanics, who have accounted for 28% and 34% of deaths, respectively (population representation: 22% and 29%, respectively).”^5^

Those same communities who have been historically harmed by the well-documented history of racialized medicine and health research in the United States (such as Tuskegee and Henrietta Lacks) and subjected to the longstanding presence of implicit and explicit biases are now bearing the inordinate burden of COVID-19. Although culturally responsive care cannot solve the deeply rooted and complex problem of disparity in health care, a failure to provide culturally responsive care can most certainly worsen it. Our care of all patients, and of patients from marginalized communities in particular, will be part of the historical record of how we, as medical professionals, rose up to meet this unprecedented crisis.

But how do we provide culturally responsive care in this environment—especially given the extreme constraints on our time? Cultural assessment—indeed all of our assessments now—must be abbreviated and efficient. That said, we believe that meaningful connection can be achieved with just a few brief questions. Even if patients have few identified needs, we suggest that they will appreciate being seen and valued as individuals, rather than failing bodies, in their time of greatest need.

In addition to asking about key family members and religion/spirituality, for patients who can speak for themselves, we might also ask “Knowing that you are in isolation, are there any photos, books, music, etc. that would be comforting to you?” In the context of a conversation about goals of care, we might ask “If we knew that time were getting short—if you were nearing death—are there things that are important that we do for you? Are there people who we should make sure you can see or hear? Is there music we should play or scripture that we should make sure is read? Are there any rituals that are important to you?” For those patients who cannot speak, we might ask their families the same questions. These conversations can be helpful even if they are brief. Although many of these questions seem like simple matters of kindness or logistics, they are also important elements of individualized care that access culture, family life, and deeply held needs. Throughout our interactions, awareness of the historical and present context of health care disparities and the mistreatment of particular communities is important.

As medical professionals, and as PC providers in particular, we have a responsibility to continue to provide culturally responsive care to patients and families, especially in the face of this overwhelming pandemic. Although it may be particularly challenging, it is especially imperative in the face of isolation, mistrust, and the disproportionate impact of this illness on communities with a long history of mistreatment by the medical system. We would suggest that this be a key element of the national, regional, and local PC discourse, despite the extreme limitations of time and resources at present. The *Field Manual for Palliative Care in Humanitarian Crises* notes “cultural self-awareness, cultural humility, and cultural curiosity are essential to providing effective care, whether in the clinic or in a disaster zone.”^6^ We couldn't agree more.

## Abbreviation Used

PC: palliative care

## Notes

^*^ We have chosen to use the term “cultural responsiveness” here. Other terms commonly utilized in the literature include cultural competence, cultural awareness, cultural sensitivity, cultural curiosity, and cultural humility. A complete discussion of the utility of each term is outside of the scope of this manuscript.^1^
